# Supplementary material for: Leveraging neighborhood representations of single-cell data to achieve sensitive DE testing with miloDE
Source: Genome Biol. 2024 Jul 18;25:189. doi: 10.1186/s13059-024-03334-3 (PMC11256449; doi:10.1186/s13059-024-03334-3)
Supplement: Supplementary file 1 — Additional file 1: Supplementary Notes. [file 13059_2024_3334_MOESM1_ESM.docx]

**Supplementary Note 1.** ‘**Supervised’ embedding enables more sensitive detection of DE genes.**

Given the range of integration/embedding techniques available [[91,92]](https://paperpile.com/c/Lu4Yr9/GWEt+DmYa), it is important to consider how they might impact the detection of DE genes. Such techniques aim to group cells that are transcriptionally similar, while DE approaches aim to identify genes that are transcriptionally distinct. Given that miloDE operates on embeddings, it might appear futile to attempt to identify differentially expressed genes. However, this paradox can be at least partially resolved by specifying that the task of miloDE is to identify case-specific cell states (defined as a change in expression of certain gene(s) in the given region(s) of the manifold) that are not present in the control data. Within this paradigm, we can decompose the contribution of each gene to the final manifold into a control-specific (or shared) component (i.e. how much this gene contributes to the formation of the cell types in both control and case settings) and a case-specific component (manifestation of the gene’s relevance only in the case setting). Therefore, to maximise the power to identify a per gene case-specific component and determine whether it is significant, we want to minimise the influence of the case-specific signal in any given embedding. Driven by this, we reasoned that ‘supervised’ approaches, where models are learned on the reference (i.e. control) data followed by the transfer of the query (i.e. case) data using the learned model (e.g. Azimuth [[4]](https://paperpile.com/c/Lu4Yr9/zl7T) and scArches [[5]](https://paperpile.com/c/Lu4Yr9/C0Z5)), would be more suitable than ‘unsupervised’ integration of both reference and query data (e.g. MNN [[34]](https://paperpile.com/c/Lu4Yr9/lZG2), Harmony [[93]](https://paperpile.com/c/Lu4Yr9/ZvhX), scVI [[35]](https://paperpile.com/c/Lu4Yr9/cUxa), etc), albeit the performance of the latter group will depend on whether query-specific genes are included in the set of informative features used for integration. Specifically, we hypothesise that if we use a ‘supervised’ embedding, differentially expressed case-specific genes will be called as DE, whereas changes in expression (across the manifold) of genes that contribute to the shared variance (e.g. cell type markers) will more likely lead to DA. Accordingly, performing a joint analysis using both Milo [[30]](https://paperpile.com/c/Lu4Yr9/O8zD) and miloDE will allow more in-depth characterisation of genes that are alternatively regulated upon a condition. By contrast, for the ‘unsupervised’ embeddings, DE of sufficient effect size in both case-specific genes and genes contributing to shared variance might lead to DA instead of DE, thus confounding a true estimate of DA and impeding our capacity to detect DE.

To formally assess this, we performed simulations to assess how DE in both case-specific and ‘shared’ genes contributes to DA and DE (**Supplementary Note 1, Fig. 1**). Specifically, we first generated a base simulation, in which we assigned cells to two conditions; within each condition 10% of genes are set to be DE in order to simulate two cell types (**Supplementary Note 1, Fig. 1A**). These genes are then referred to as genes contributing to the shared variance or ‘shared’ genes. While in the base simulation there is no DE between the conditions, we also performed a series of *in silico* perturbations, by altering the expression for the cells from one cell type (Cell type 1) and one condition (condition B) only (see Methods). Specifically, we either altered only the expression of genes that contribute to the shared variance (for all shared genes, but with varying effect size) or only some of the remaining genes (varying number of genes) or both. Additionally, to simulate a biologically meaningful scenario, we controlled DE in shared genes in such a way that it will essentially result in cells from Cell Type 1, condition B acquiring the phenotype similar to Cell Type 2. Since the motivation behind using the ‘supervised’ embedding is to minimise the input from case-specific genes, we approximated ‘supervised’ and ‘unsupervised’ embeddings by either excluding or including genes that do not contribute to the shared reference. In total, we performed 3 independent rounds of perturbations, and in each round, we performed 99 perturbations in total by varying the effect size of DE for ‘shared’ genes and the number of ‘not shared’ DE genes. When examining results obtained from the ‘supervised’ embedding, DA depends solely on the effect size and not the number of ‘not shared’ DE genes, whereas for the ‘unsupervised’ embeddings, it depends on both parameters (**Supplementary Note 1, Fig. 1B**). In addition, in the ‘unsupervised’ case, DE in ‘not shared’ genes drives a separation in the transcriptional space between conditions A and B for Cell Type 1, thus impeding the detection of those genes as DE, especially when the number of those genes is high (which leads to a stronger separation) (**Supplementary Note 1, Fig. 1C**). Overall, we conclude that ‘supervised’ embedding results in a more biologically appropriate ‘mixing’ of cells and provides a better platform to estimate DE in case-specific genes.

Finally, we sought to quantitatively estimate the impact of different embedding schemes on the performance of miloDE in real data. We analysed transcriptional changes occurring in chimeric mouse embryos, in which tdTomato+ mouse embryonic stem cells (no knock out) were injected into wild type blastocysts [[38]](https://paperpile.com/c/Lu4Yr9/kfGF). We used an atlas of Wild Type (WT; i.e. non-chimeric) gastrulating mouse embryos as a “control” (henceforth referred to as WT), and cells from the chimeric embryos were used as the “case” (henceforth referred to as ChimeraWT). Thus, we tested how the presence of foreign embryonic stem cells affects ChimeraWT cells (when compared to ‘true’ WT cells). In total, we computed 16 embeddings: we used 4 different approaches (Azimuth [[4]](https://paperpile.com/c/Lu4Yr9/zl7T), MNN [[34]](https://paperpile.com/c/Lu4Yr9/lZG2), scVI [[35]](https://paperpile.com/c/Lu4Yr9/cUxa) and scANVI [[75]](https://paperpile.com/c/Lu4Yr9/s5Wj)) and for each of the methods, we used a ‘supervised’ and ‘unsupervised’ version (see Methods) as well computing the embedding using highly variable genes (HVGs) estimated from either only WT (i.e. control) or from both WT + ChimeraWT cells (**Supplementary Note 1, Fig. 2A**). The motivation for such setup was driven by the rationale that in practice we need ‘supervised’ embedding because we want to minimise the input from case-specific genes, and in some cases, this input can be minimised by ensuring that case-specific genes are excluded from the HVG selection. Additionally, to estimate whether the difference between the embeddings is robust with respect to a certain degree of stochasticity driven by a semi-random selection of index cells (and therefore a stochasticity in a neighbourhood assignment), for each embedding, we performed 5 independent neighbourhood assignments (see Methods). To identify embedding methods that minimise the case-specific variance (in this case the chimera-specific variance), we selected 3 genes that are broadly upregulated (i.e. across the majority of cell types) in the ChimeraWT cells with a broad range of the effect size (**Supplementary Note 1, Fig. 2B**), and used the standard deviation in expression across cells in a neighbourhood to assess the homogeneity of each neighbourhood. We hypothesised that in embeddings where the contribution of chimera-specific variance to the latent embedding model (and accordingly the neighbourhood assignment) is minimised, expression distributions for these genes across chimera cells from the same neighbourhoods would be randomly sampled and contain both high and low expression values. On the other hand, if chimera-specific variance contributes to the latent embedding, it is more likely that these genes contribute to the neighbourhood assignment, thus resulting in neighbourhoods that, while otherwise similar, differ in the average expression of chimera-specific genes. As expected, since the genes we selected are chimera-specific, we observe that the standard deviation for all of genes is systematically (albeit marginally for some genes) lower for the ‘unsupervised’ embeddings in which HVGs were calculated from both control and case cells (in other words, embeddings with a contribution from case-specific genes) (**Supplementary Note 1, Fig. 2C**). This difference is consistent across 5 independent neighbourhood assignments, thus suggesting that the type of embedding and not the neighbourhood assignment itself is a driving factor for the segregation of cells into more or less homogenous neighbourhoods (**Supplementary Note 1, Fig. 2D**). The only method for which this does not hold is Azimuth. We hypothesise that this is due to the specifics of the anchor-based approach employed by Azimuth [[76]](https://paperpile.com/c/Lu4Yr9/8syk), in which pairwise correspondences between cells from different conditions (termed anchors) are used to perform the integration, and it is likely that the case-specific genes do not contribute greatly for the anchor identification (since they are only relevant for some of the samples). Additionally, we sought to assess how these properties of different embeddings impact DE detection. Intriguingly, we observe a systematically lower effect size for the ‘unsupervised’ embeddings in which HVGs were calculated from both control and case cells (**Supplementary Note 1, Fig. 2E, F**). Importantly, Log Fold Changes (logFC) for the other types of embeddings generally coincided with the pseudo-bulk estimate (single DE test for all cells grouped together, **dashed red line**, **Supplementary Note 1, Fig. 2E, F**), thus suggesting that in embeddings with a contribution from case-specific variance the effect size is indeed underestimated. Finally, we calculated the fraction of significantly DE neighbourhoods. We observe that while there is virtually no difference for *Rpgrip1* and *Cbx3*, for *Actb* - the gene with the highest base expression in WT cells - the detection rate for ‘unsupervised’ embeddings in which HVGs were calculated from both control and case cells is slightly lower (**Supplementary Note 1, Fig. 2G, H**).

Overall, our analysis suggests that ‘supervised’, reference-based embeddings will yield a more sensitive DE detection and therefore are more suitable for miloDE implementation.

**Figures.**

**Supplementary Note 1, Figure 1.** **Transcriptional shifts in case-specific genes lead to DA in ‘unsupervised’, but not ‘supervised’ embeddings.**

1. Cartoon representing simulations that are used to assess how DE in case-specific and shared genes leads to DA in ‘unsupervised’ and ‘supervised’ embeddings. In a base simulation (top UMAP), we have two ‘identical’ conditions, and within each condition, we have two cell types (driven by 10% of genes being DE between the cell types). We then perform perturbation in Cell Type 1 of condition B, in which we either alter counts only for ‘not shared’ genes (left bottom UMAP), ‘shared’ genes (middle bottom UMAP), or both (right bottom UMAP).
2. Heatmaps representing the extent of DA in each perturbation and each embedding (‘unsupervised’ = PCA on all the genes, ‘supervised’ = PCA on ‘shared’ genes).
3. Heatmaps representing the extent of DE (average across all ‘not shared’ DE genes) in each perturbation and each embedding.

**
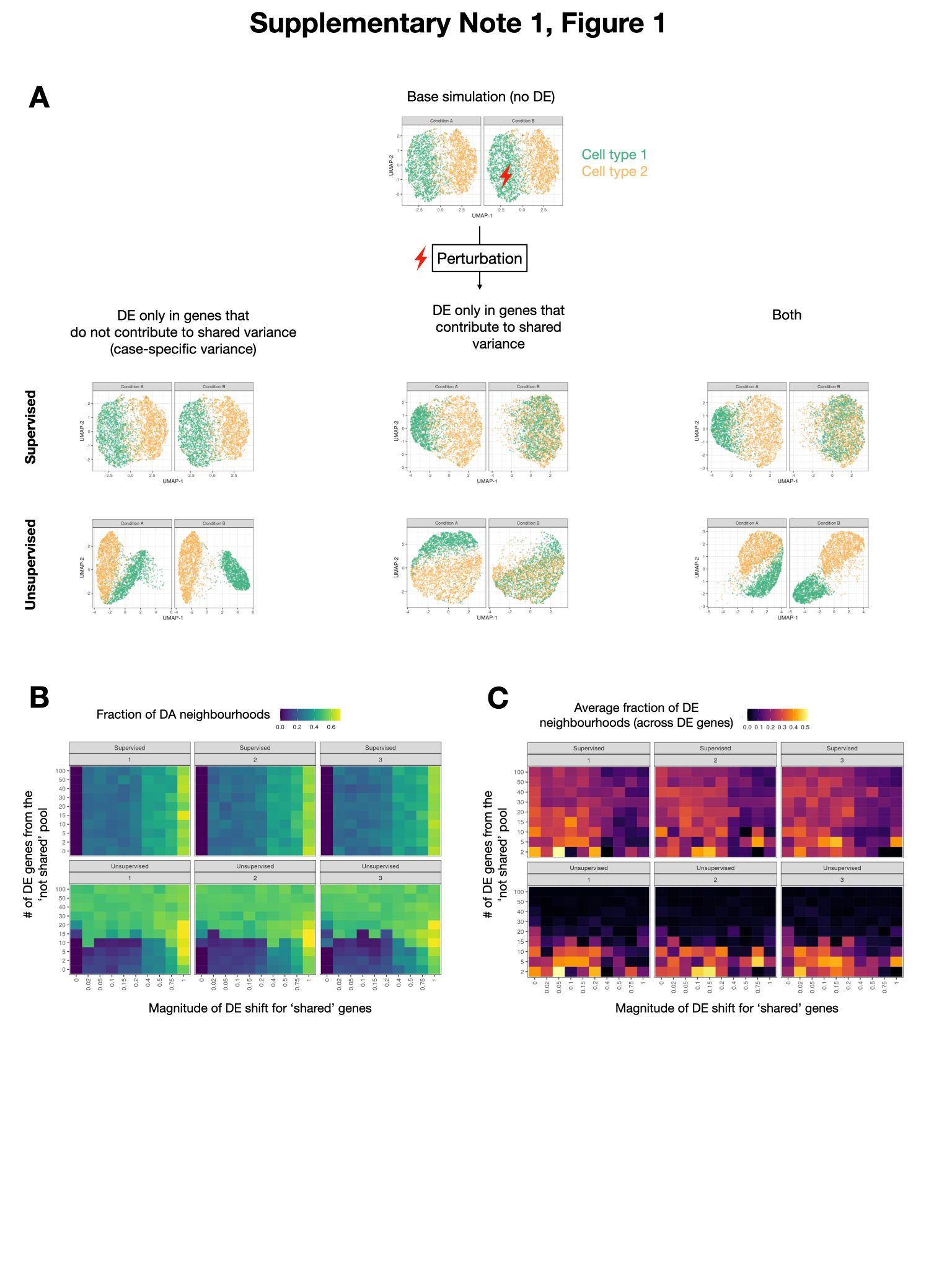
**

**Supplementary Note 1, Figure 2.** **Reference-based ‘supervised’ embedding schemes yield higher sensitivity for DE identification.**

1. Description of calculated embeddings.
2. Boxplots of selected chimera-specific genes, representing the expression across cells. Each facet corresponds to one gene, colours correspond to the condition.
3. Boxplots representing within neighbourhood distribution of standard deviations within the neighbourhoods for the selected genes (y-axis) for a single neighbourhood assignment. Colours and x-axis correspond to different embedding schemes, and facets correspond to the condition. Red asterisks represent significant differences (* < 0.05; *** < 0.001, Welch’s T-test).
4. Boxplots representing within neighbourhood distribution of average standard deviations within the neighbourhoods for the selected genes (y-axis) (across 5 independent neighbourhood assignments). Colours and x-axis correspond to different embedding schemes, and facets correspond to the condition.
5. Boxplots representing the logFC distribution for the selected genes for a single neighbourhood assignment. Colours and x-axis correspond to different embedding schemes, and facets correspond to the condition. Red dashed lines correspond to logFC from the pseudo-bulk estimate. Red asterisks represent significant differences (** < 0.01; *** < 0.001, Welch’s T-test).
6. Boxplots representing the average logFC distribution for the selected genes (across 5 independent neighbourhood assignments). Colours and x-axis correspond to different embedding schemes, and facets correspond to the condition. Red dashed lines correspond to logFC from the pseudo-bulk estimate.
7. Barplots representing the fraction of significantly DE neighbourhoods for a single neighbourhood assignment. Colours and x-axis correspond to different embedding schemes, and facets correspond to the condition.
8. Boxplots representing the average fraction of significantly DE neighbourhoods (across 5 independent neighbourhood assignments). Colours and x-axis correspond to different embedding schemes, and facets correspond to the condition.

**
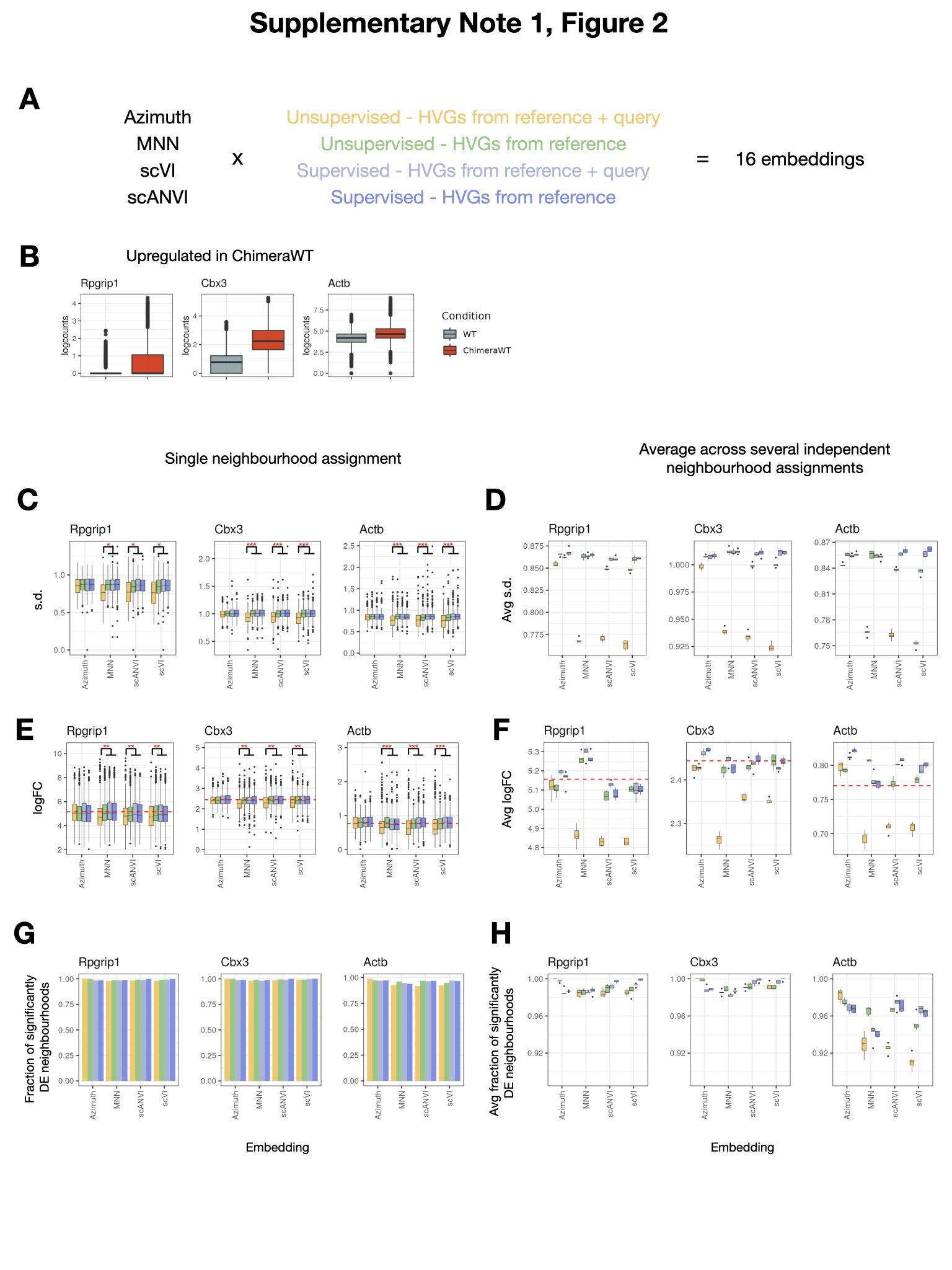
**

**Supplementary Note 2.** **Importance of a sufficient number of cells for sensitive DE detection.**

Since the power to detect DE using edgeR is highly dependent on the number of tested cells [[14]](https://paperpile.com/c/Lu4Yr9/uE34), it is important to ensure that the neighbourhood assignment method results in neighbourhood sizes (i.e. number of cells per neighbourhood) that are large enough to enable sensitive and specific DE detection. To this end, we performed simulations using splatter [[73]](https://paperpile.com/c/Lu4Yr9/S0bl) where we varied the total number of replicates as well as the imbalance between control and case replicates, and the expected effect size (logFC) (**Supplementary Note 2, Fig. 1**). We then performed 2000 downsamplings for each dataset, in which we sampled between 50 and 3000 cells, followed by estimation of DE detection for each downsampled sub-dataset.

As expected, sensitivity and FDR are highly dependent on the number of cells and replicates tested (**Supplementary Note 2, Fig. 1A, B**). Specifically, for all simulated datasets (i.e. varying numbers of control and case replicates), the average number of cells per replicate and total number of replicates appear to be the dominant factors, with sensitivity being positively scaled with the average number of cells per replicate and number of replicates (**Supplementary Note 2, Fig. 1A, second and fourth columns**). While controlling for the total number of cells, we observe that the number of replicates and class imbalance affect DE detection as well. Specifically, higher class imbalance results in lower sensitivity, suggesting that the minimum number of cells (across the conditions) is the bottleneck for sensitivity in DE detection (**Supplementary Note 2, Fig. 1B**). Intriguingly when the number of control and case replicates is the same, the total number of cells across all replicates is a determining factor for DE detection (**Supplementary Note 2, Fig. 1A, first column**). Indeed, while controlling for the total number of cells, we observe that the difference between datasets with a variable number of replicates (for datasets with a balanced number of replicates across two conditions) is modest (**Supplementary Note 2, Fig. 1A, inlet**). This can be explained by the absence of the batch effect in the introduced simulations leading to the marginal variance across replicates when compared to the variance between cells within each replicate (which is particularly high for the pseudo-bulks consisting of fewer cells). In turn, when the variance within the replicates is much greater than the variance across the replicates, the low detection rate for small pseudo-bulks can not be rescued by the higher number of replicates. Accordingly, when introducing batch effect between replicates (thus increasing variance across replicates), we observe a higher impact of different numbers of replicates on DE detection (**Supplementary Note 2, Fig. 1C**). Finally, we note that if the detection of DE genes is defined solely by the p-value, FDR plateaus around 0.15 and remains high across a wide range of the total number of cells (**Supplementary Note 2, Fig. 1A, first and second columns**). To retrieve this, we suggest that DE detection needs to be defined by both the p-value and the expected to be detected minimum effect size (**Supplementary Note 2, Fig. 1A, third and fourth columns**).

Importantly, sensitivity to detect DE genes with logFC=1 is, on average, below 0.65 if the number of tested cells is below 350 (~0.85 for logFC = 2, **Supplementary Note 2, Fig. 1A, C and inlet**). Therefore, assuming that 0.65 is the target sensitivity and logFC = 1 is the target effect size, we set 350 cells per neighbourhood as the target neighbourhood size. In the original Milo approach [[30]](https://paperpile.com/c/Lu4Yr9/O8zD), the kNN graph is used to represent the relationship between cells, and the parameter *k* (i.e. number of nearest neighbours) controls the average neighbourhood size. When tested on the mouse chimera dataset (the same dataset used for the embedding analysis, **Supplementary Note 1**), we observed a linear relationship between *k* and average neighbourhood size: to reach an average neighbourhood size above 350, *k* needed to be above 200 (**Supp. Fig. 1**). More generally, while the precise relationship between *k* and neighbourhood size distribution will depend on the dataset in question, the nearly linear dependence will force the optimal range for *k*, on average, to be on the order of hundreds. However, as *k* increases, the homogeneity of the neighbourhoods will inevitably decline, potentially leading to rare cell types (defined as those present in considerably fewer quantities than the ideal neighbourhood size) being absorbed into transcriptionally related, but more abundant cell types. To address this problem, we propose using a 2nd-order kNN graph, where we first compute the standard kNN graph (henceforth referred to as the 1st-order kNN graph), followed by assigning edges between any two cells that have at least one common neighbouring cell. To assign neighbourhoods, for each selected index cell (see Methods), we assign all cells connected with it to a single neighbourhood. As *k* increases, the average neighbourhood size increases considerably faster than it does for the 1st-order KNN graph, with a target size of 350 being achieved when *k* is 20-25 (**Supp. Fig. 1, right panel**). Most importantly, we suggest that with 2nd-order optimization, we achieve higher neighbourhood homogeneity while controlling for average neighbourhood size. In other words, for abundant cell types that contain many transcriptionally similar cells, the method will result in sufficient neighbourhood sizes. On the other hand, for rare cell types, neighbours of neighbours will mostly lie within the same cell type, and thus the neighbourhoods will be smaller, and thus considerably more homogeneous compared to the 1st-order kNN graph.

We list DE detection results for the analysed simulations and downsamplings in **Supplementary Table 1**.

**Figures.**

**Supplementary Note 2, Figure 1.** **Sensitivity in DE detection scales together with the number of replicates and number of tested cells.**

1. Trends representing the relationship between the total number of cells (x-axis, first or third column) or the average number of and sensitivity (top row), specificity (middle row), FDR (bottom row). Each line corresponds to one simulation; simulations vary by the total number of replicates (in colour), number of control replicates is the same as the number of case replicates. DE detection is either defined using p-value solely (first and second columns) or using p-value in combination with the minimum absolute logFC (>= 0.5) required (third and fourth columns). The inlet representing a zoomed region from the top left panel.
2. Similar to A, but number of control and case replicates is varied (with the total number = 12 across all simulations), and colour corresponds to the ratio between the number of control and case replicates.
3. Trends representing the dependence of sensitivity (y-axis) on the total number of cells (x-axis) and effect size (facets, in columns) and presence of batch effect (facets, in rows). Note, that simulations in A) and B) are all performed in the absence of batch effect, with target logFC = 1. As in A), simulations vary by the total number of replicates (in colour), number of control replicates is the same as the number of case replicates.

**
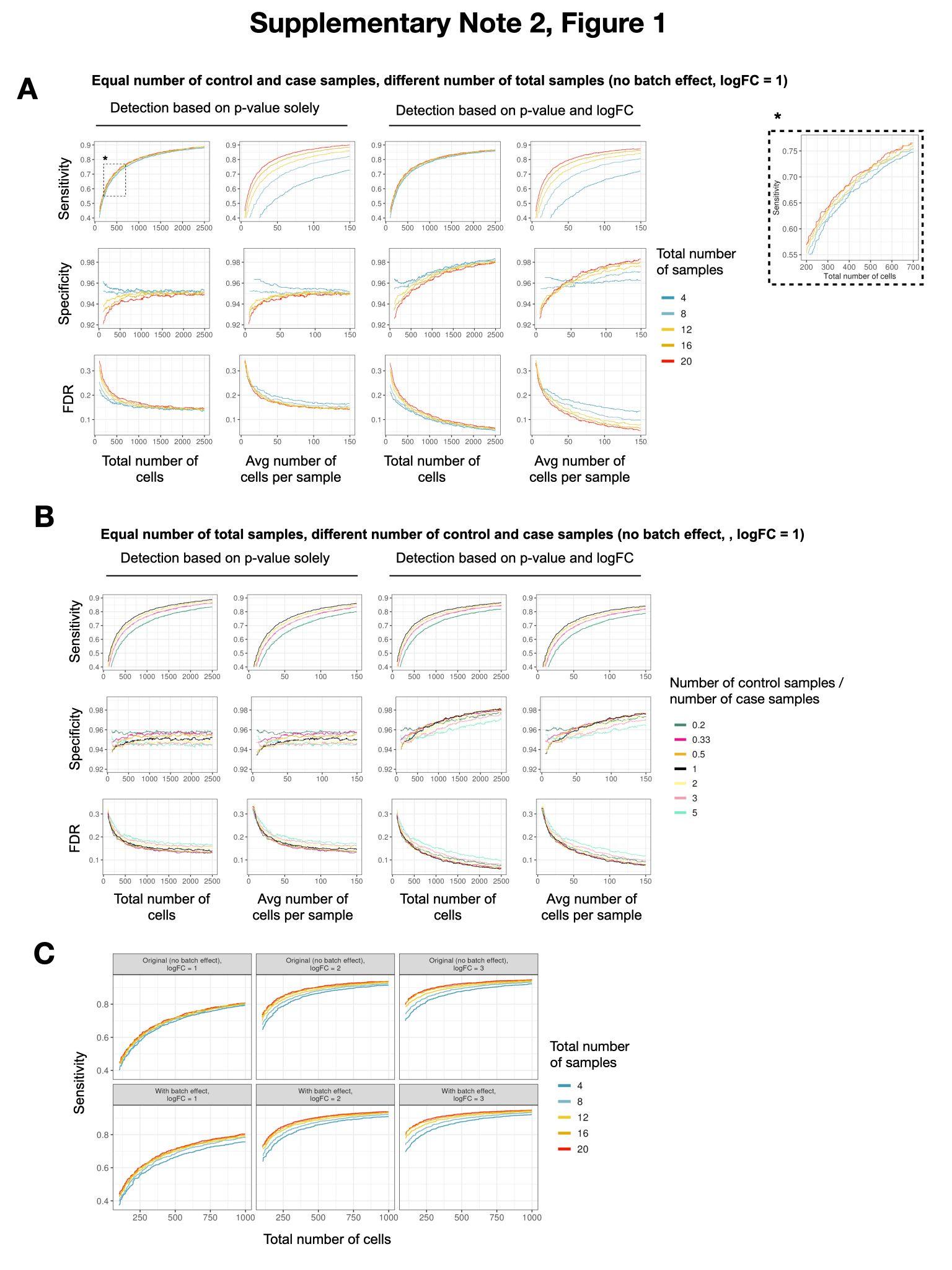
**

**Supplementary Note 3.** **A priori identification of ‘unperturbed’ neighbourhoods decreases the number of tested neighbourhoods and reduces the burden of multiple testing correction.**

To decrease the burden of multiple testing correction, a standard practice in DE analysis is to discard genes that are lowly expressed in both case and control samples. We suggest that a similar procedure can be applied to exclude neighbourhoods from testing. To this end, we adapt the classifier algorithm Augur whose original purpose is the ranking of the cell types by the degree of their perturbation in case-control studies ([[71]](https://paperpile.com/c/Lu4Yr9/gn1U), Methods). In brief, Augur builds Random Forest-based classifiers to distinguish case and control cells and returns the AUC of the classifiers. We implement Augur to return the AUC for each neighbourhood, and the user can select the AUC threshold to decide which neighbourhoods will be further supplied for DE testing (recommended default is 0.5).

To test how AUC distribution depends on whether DE is present between two tested groups, we used the R package splatter that simulates scRNA-seq counts with the desired properties. Specifically, we generated several datasets with different number of genes that are DE (including 0 as a control), different effect sizes, and introduced batch effect (Methods). Our simulations confirm that in comparisons where DE exists, AUCs are consistently higher than 0.5, whereas, in the absence of the DE, AUCs are driven by the existence of batch effects between case and control samples (with AUCs centering around 0.5 in datasets with no or balanced batch effect) (**Supplementary Note 3, Fig. 1**). Therefore, we suggest that while it is beneficial to discard uninteresting neighbourhoods to minimise the computing time and the burden of multiple testing correction, the practical benefit of this step in the datasets with complex batch effects is negligible, and we leave this optional step to the decision of the user. In the implementation of this step in the package, we return AUC calculated for each neighbourhood, and subsequently, the user can select their own AUC threshold.

**Figures.**

**Supplementary Note 3, Figure 1.** **Random Forest based classifiers successfully identify neighbourhoods with DE as ‘important’.**

Boxplot representing the relationship between AUC (y-axis) of Random Forest classifiers and fraction of genes with DE (x-axis), effect size (in colour) and the existence of the batch effect (in facets). Red dashed line corresponds to AUC = 0.5 which we use to define neighbourhoods as relevant or irrelevant for DE testing.

**
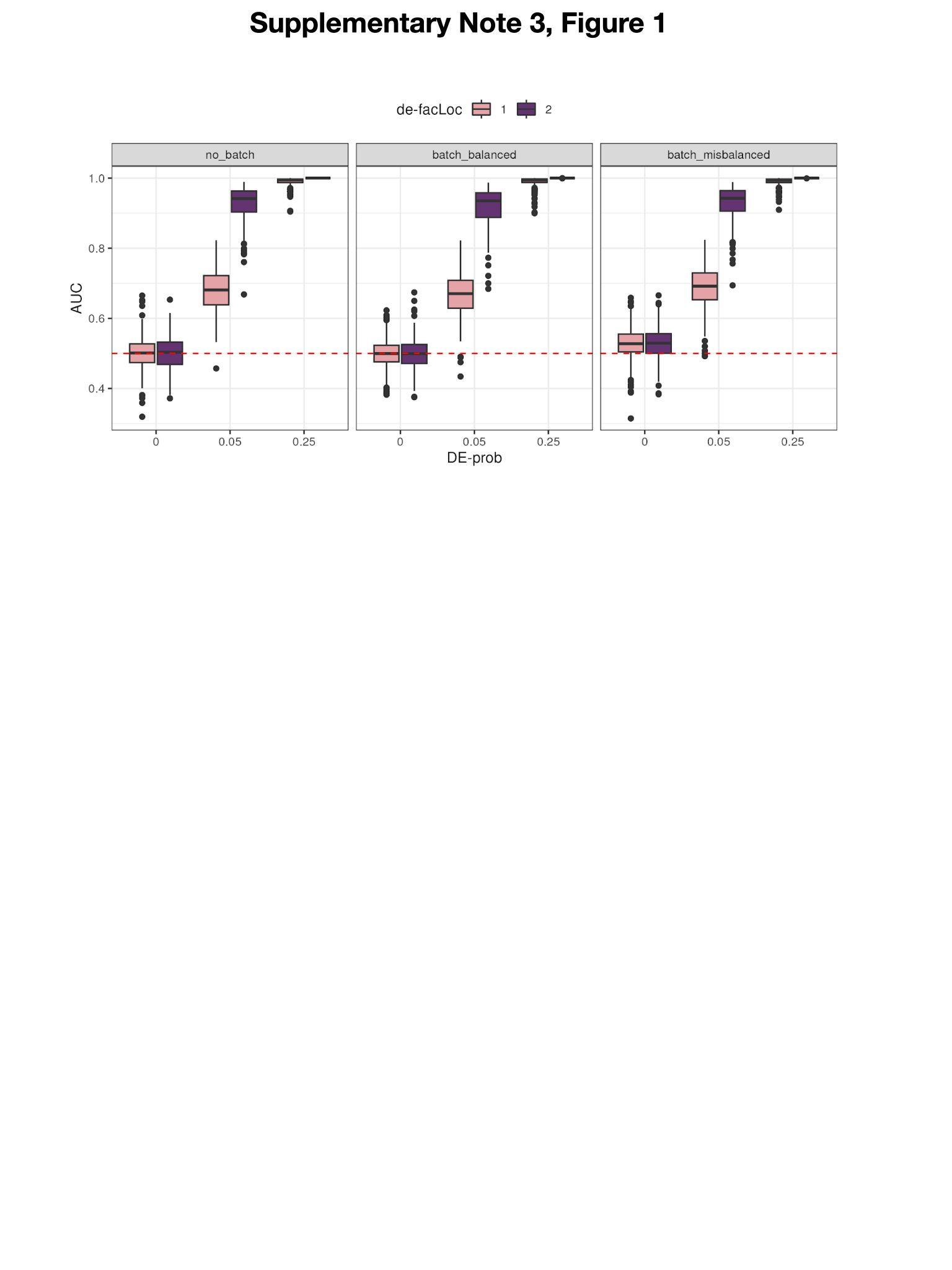
**
