# Supplementary material for: Leveraging neighborhood representations of single-cell data to achieve sensitive DE testing with miloDE
Source: Genome Biol. 2024 Jul 18;25:189. doi: 10.1186/s13059-024-03334-3 (PMC11256449; doi:10.1186/s13059-024-03334-3)
Supplement: Supplementary file 3 — Additional file 3: Supplementary Figures. [file 13059_2024_3334_MOESM3_ESM.docx]

**Supplementary Figures.**

**Fig S1. The estimate of the scaling between *k* (x-axis) and the neighbourhood size distribution (y-axis) for either 1st- or 2nd-order KNN graphs (in facets and colour).**

The dashed black line corresponds to y = 350.


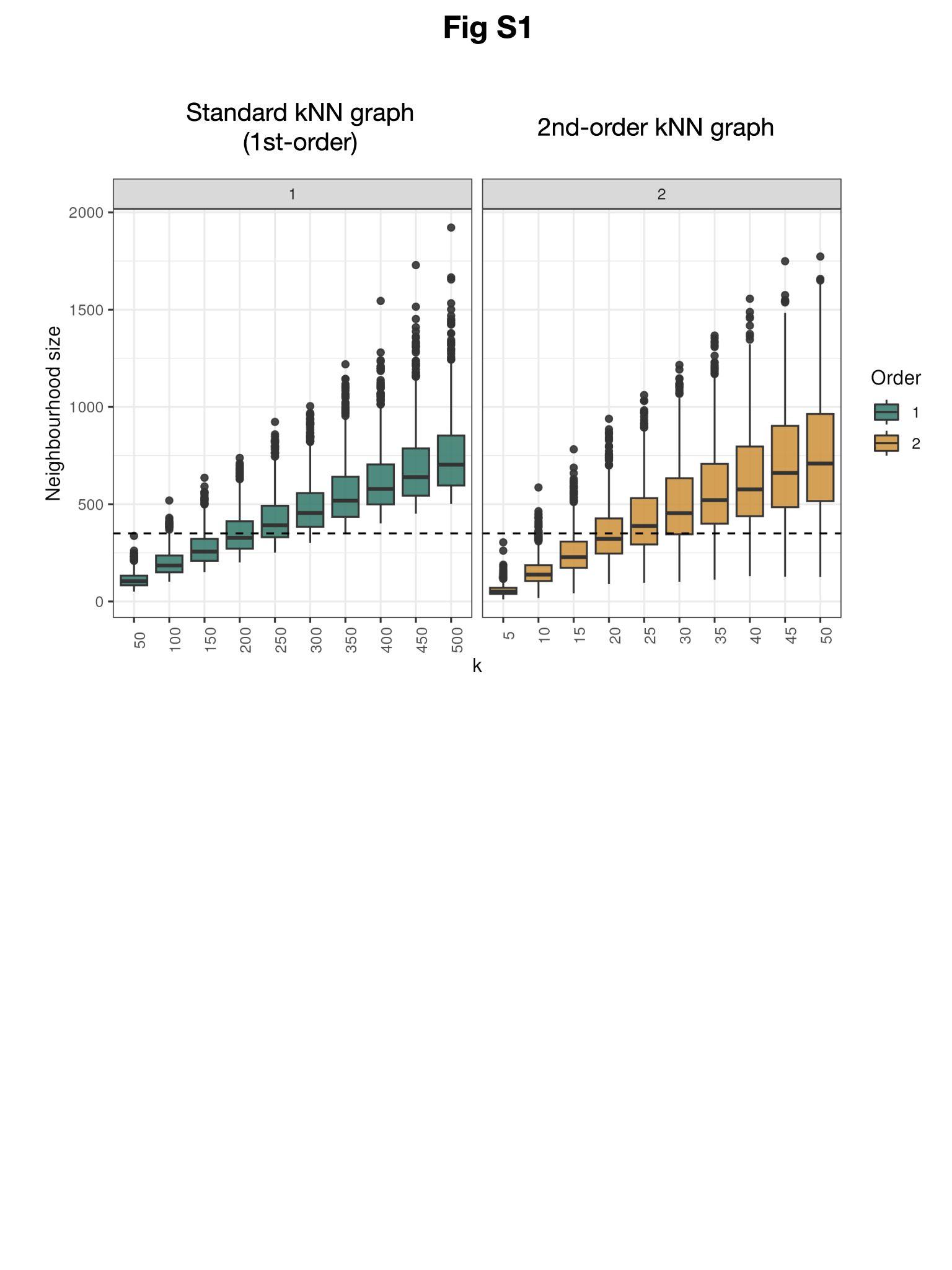


**Fig S2. 2nd-order neighbourhood graphs preserve neighbourhood homogeneity better while also controlling for the average neighbourhood size.**

1. Boxplot representing the relationship between average neighbourhood size and the selected *order-k* grid that we used to perform neighbourhoods assignments.
2. Barplot representing cell types composition across cell types and conditions (top row corresponds to the fraction of cells and bottom row corresponds to the log10 of the number of cells).
3. Boxplots representing the distribution of relative cell type enrichment scores (y-axis) across neighbourhoods for each cell type (in facets) and each neighbourhood assignments (x-axis). Colours correspond to the order of the graph.
4. Boxplots representing distribution of maximum cell type purity (across neighbourhoods) scores (y-axis) for each cell type (in facets) and each neighbourhood assignment (x-axis). Colours correspond to the order of the graph.


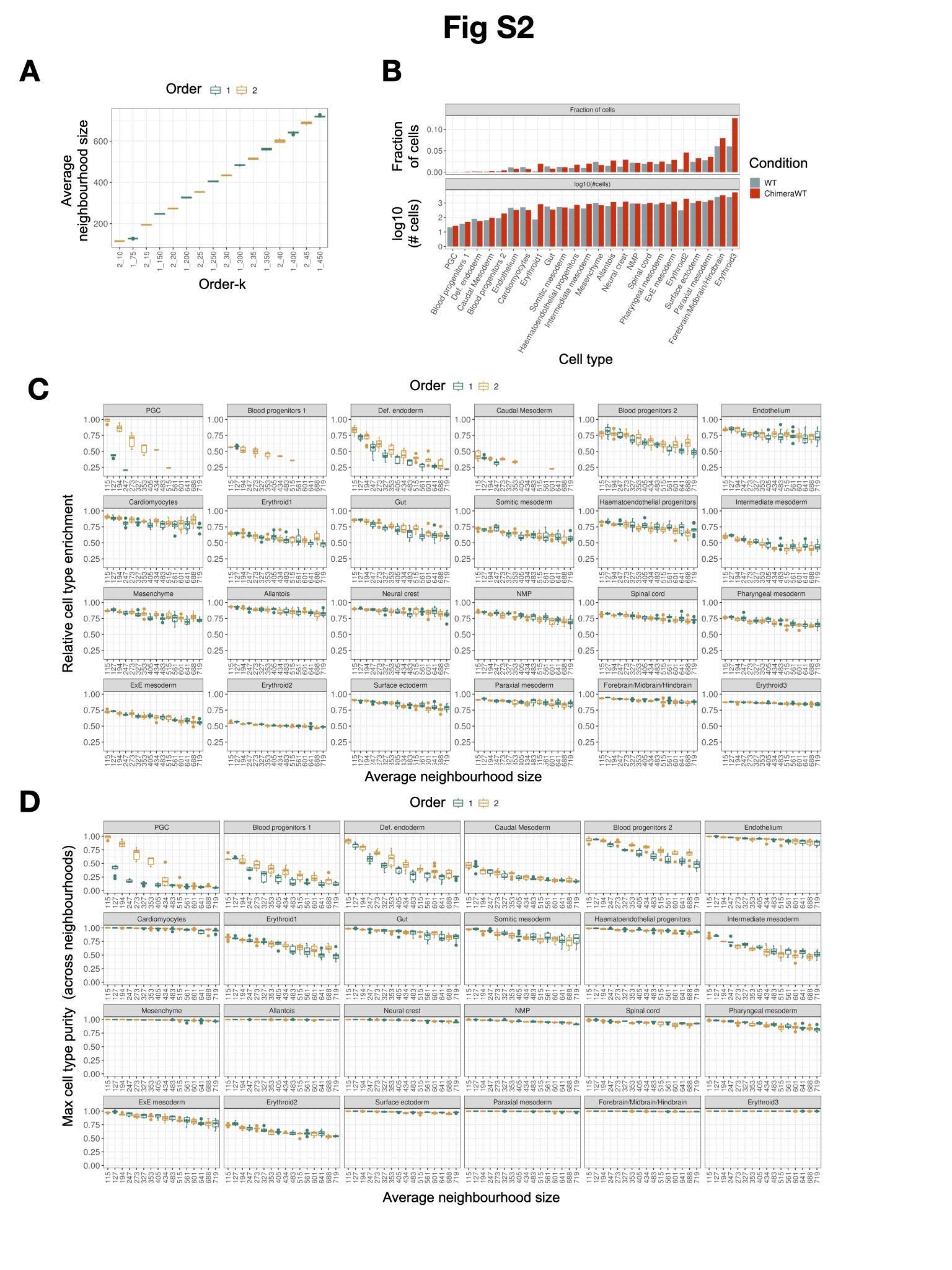


**Fig S3.** **DE detection for rare cell types is higher for 2nd-order kNN graphs.**

1. Scater plots representing the relationship between cell type purity (x-axis), number of cells from the cell type (y-axis) and DE detection power (in colour). Each point corresponds to one neighbourhood.
2. Boxplots representing the relationship between an assignment (i.e. *order-k*) and maximum DE detection power (across neighbourhoods).

**
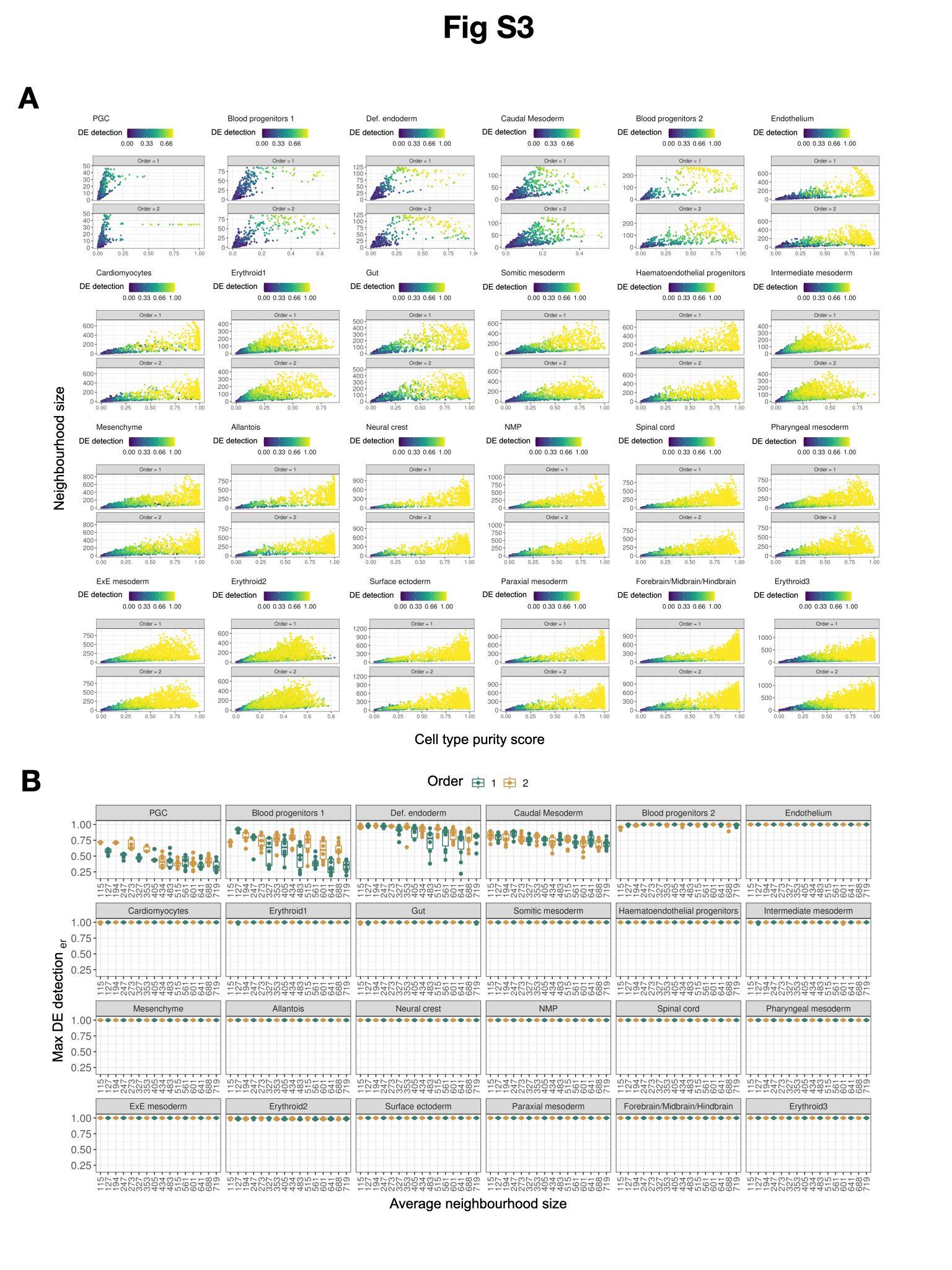
**

**Fig S4. Post hoc neighbourhood filtering minimises the number of assigned neighbourhoods while also controlling for the inclusion of all cells in at least one neighbourhood.**

Each facet corresponds to a different *k* in the graph assignment (using 2nd-order graph), and colours correspond to whether filtering was performed or not. The top panels correspond to the number of neighbourhoods that are matched between two filtering options, and the bottom panels correspond to the fraction of cells that are not assigned with any neighbourhoods.

**
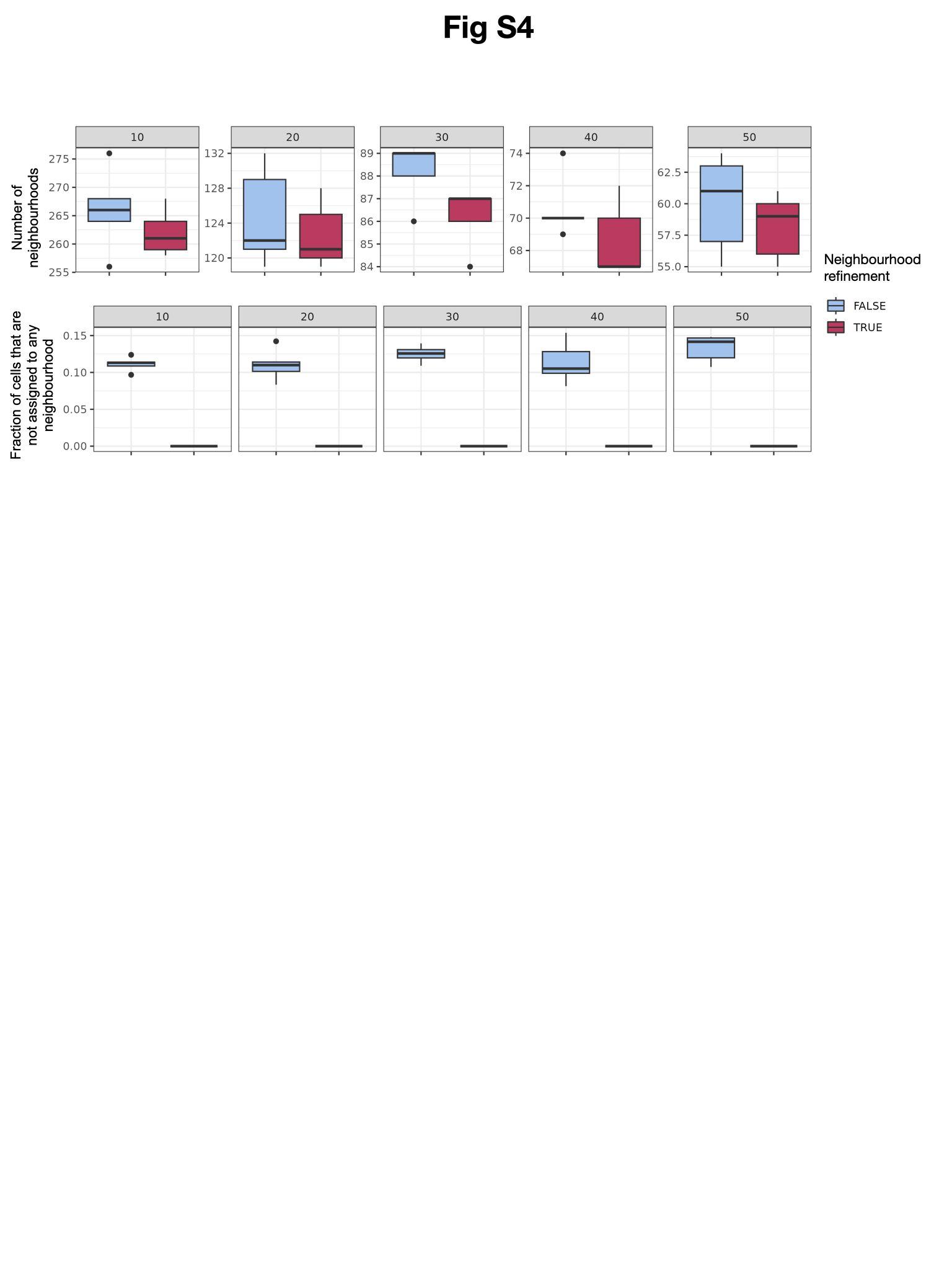
**

**Fig S5. Overview of the simulations containing two transcriptionally similar cell groups within a population.**

1. Schematic representing the simulation experiment and DE detection in miloDE and Cacoa. First, we create two cell groups imitating two sub-cell types (left panels, single-cell representation on the top, and neighbourhood representation on the bottom). We denote one group as the ‘perturbed group’ meaning that we will simulate DE specifically in this group. In the second step, we alter counts in the case cells from the perturbed group only, thus generating DE specific to the perturbed group only (middle panels). Finally, the detection of miloDE and Cacoa will be estimated on a gene basis, based on corrected across neighbourhoods p-values for miloDE (right top panels) and based on the raw or adjusted z-scores for Cacoa (right bottom panels).
2. Overview of 7 simulated meta-datasets, with varying fractions of perturbed cells (signified in the title of the corresponding panels). Each dataset contains 5 control and 5 case replicates. For each meta-dataset, we plot UMAP representation, in which colours correspond to the group identity (left panels), and neighbourhood representation, in which colours correspond to the fraction of cells from the more abundant group in the neighbourhood, size corresponds to the neighbourhood size (left panels).

**
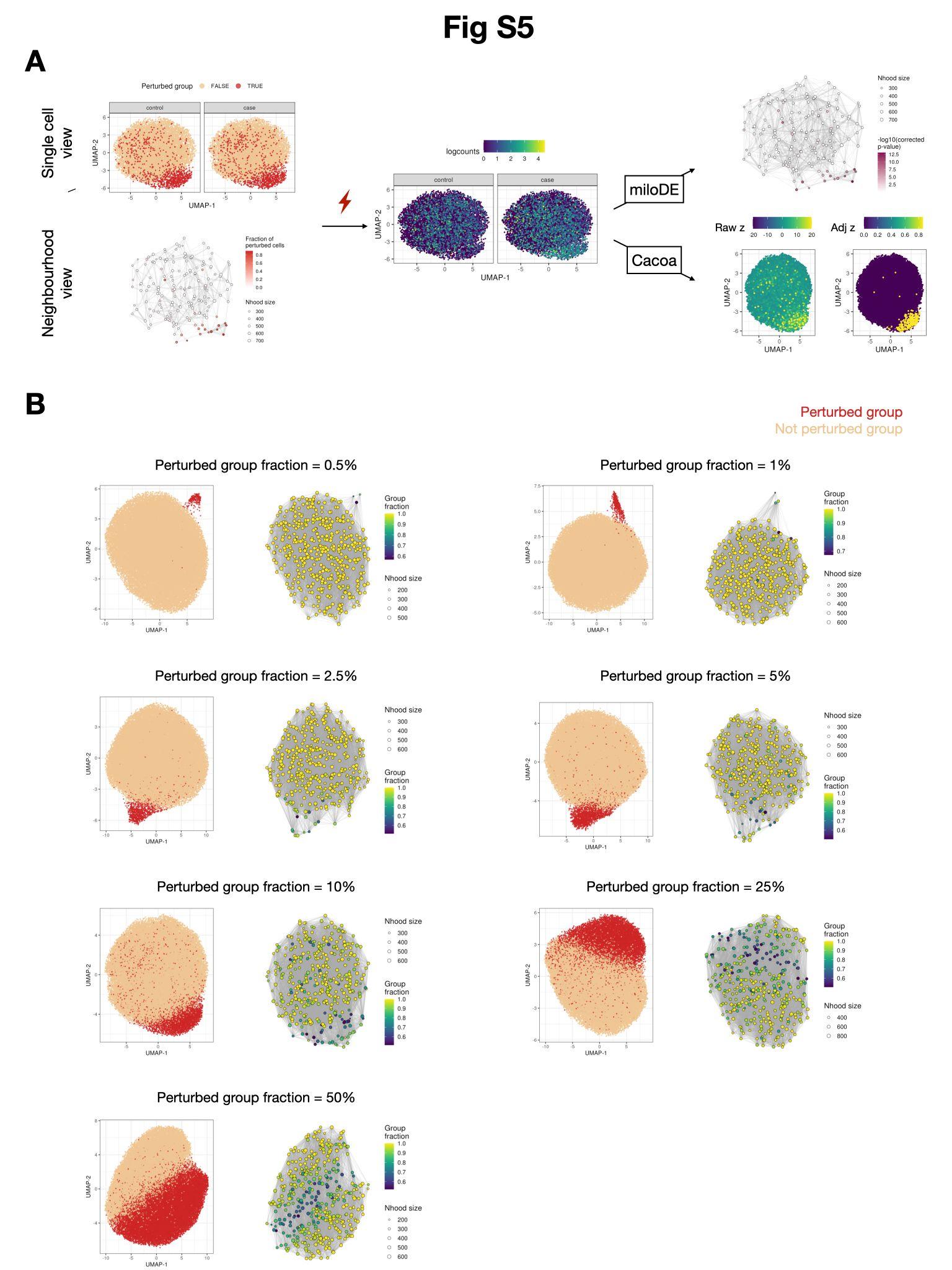
**

**Fig S6. Comparison of AUC distribution across different tested conditions.**

1. Neighbourhood-based comparison, distribution of AUCs for tests across the neighbourhoods. Ground truth ‘DE’ neighbourhoods are estimated as neighbourhoods containing the fraction of cells from the perturbed group higher than the designated purity threshold (x-axis). For each tested gene and the approach, we aggregate calculated AUC across different repetitions of the neighbourhood assignments. Note, that for miloDE, repetitions of neighbourhood assignment are grouped for each *k* separately i.e. different values of *k* in miloDE are compared as different approaches. For Cacao, using either raw or adjusted z-scores is also split into different approaches. Y-axis corresponds to AUC, colours correspond to different methods, facets correspond to different datasets, and boxplots represent a distribution across genes.
2. Single cell-based comparison, distribution of AUCs for tests across single cells. For each dataset, tested gene, and method, we aggregate calculated AUC across different repetitions of the neighbourhood assignments. Note that we further split results from Cacoa into four approaches (compared to two approaches in the neighbourhood-based comparison), based on which p-values were used and which cells were used as a ground truth DE (all cells from the perturbed group or only case cells from the perturbed group). Y-axis corresponds to AUC, x-axis corresponds to different meta-datasets, colours correspond to different methods, facets correspond to different subsamplings of the meta-datasets (to vary the number of case and control samples), and boxplots represent a distribution across genes.

**
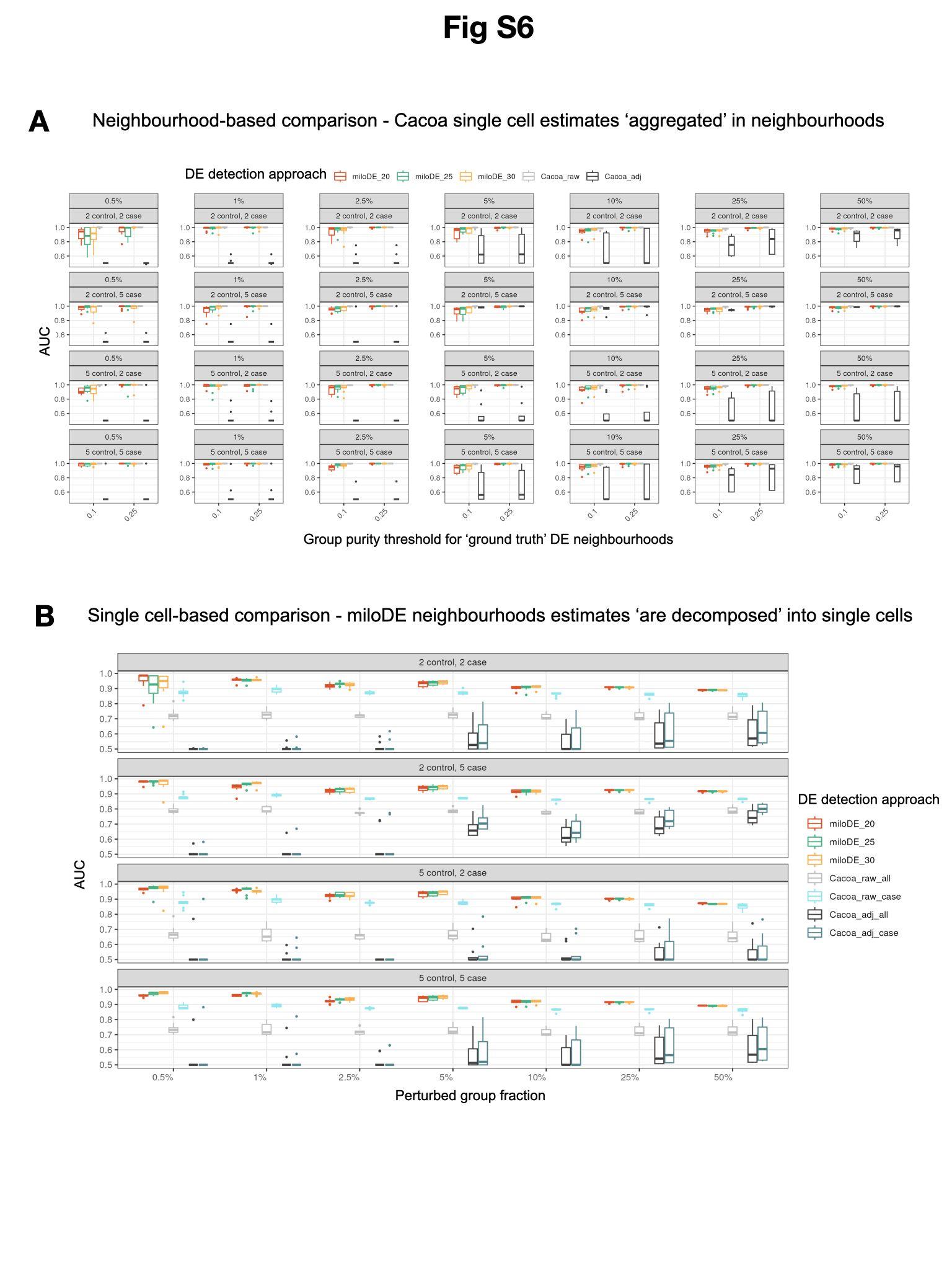
**

**Fig S7. DE detection in miloDE is robust to the stochasticity in the neighbourhood assignments.**

1. For each group purity threshold (x-axis), boxplots representing how many neighbourhoods have a fraction of perturbed cells higher than the designated purity threshold. Each colour corresponds to *k*, and each facet corresponds to the dataset. Each boxplot distribution consists of different rounds of neighbourhood assignments.
2. Boxplots representing AUC distribution for each dataset (x-axis), group purity thresholds (facets, split by columns), *k* (facets, split by rows), and tested genes (in colour). Each boxplot distribution consists of different rounds of neighbourhood assignments.

**
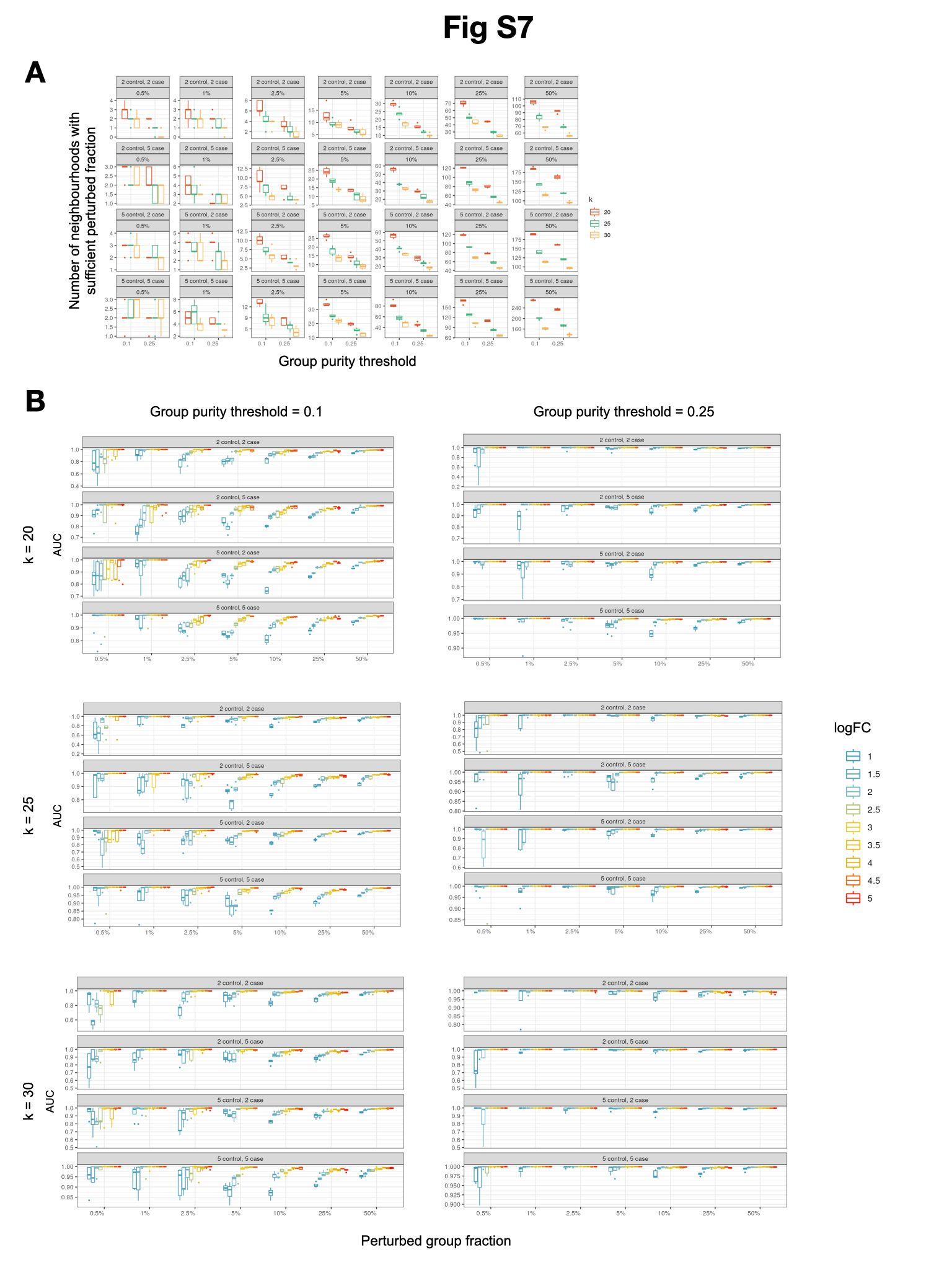
**

**Fig S8. miloDE enables more sensitive DE detection when compared to the pseudo-bulk approach.**

1. Heatmaps representing the sensitivity of DE detection for the perturbed genes using the pseudo-bulk approach or miloDE. The left panel corresponds to the pseudo-bulk approach, and the panels on the right correspond to miloDE (one panel for each *k* and group purity threshold, results are aggregated across different repetitions of the neighbourhood assignment). Facets correspond to different subsamplings of the meta-datasets (to vary the number of case and control samples), x-axis corresponds to the estimated effect size of the gene (using pseudo-bulk approach on the perturbed cells only), y-axis corresponds to the fraction of perturbed group. Sensitivity in the pseudo-bulk is a binary value (based on whether FDR < 0.1). Sensitivity for miloDE is a continuous value and is estimated as a sensitivity of the detection across the neighbourhoods.
2. Heatmaps representing the detected logFC for the perturbed genes using the pseudo-bulk approach or miloDE. The left panel corresponds to the pseudo-bulk approach, and the panels on the right correspond to miloDE (one panel for each *k* and group purity threshold, results are aggregated across different repetitions of the neighbourhood assignment). Facets correspond to different subsamplings of the meta-datasets (to vary the number of case and control samples), x-axis corresponds to the estimated effect size of the gene (using pseudo-bulk approach on the perturbed cells only), y-axis corresponds to the fraction of perturbed group. Detected logFC for miloDE is calculated as average logFC across significantly DE neighbourhoods.

**
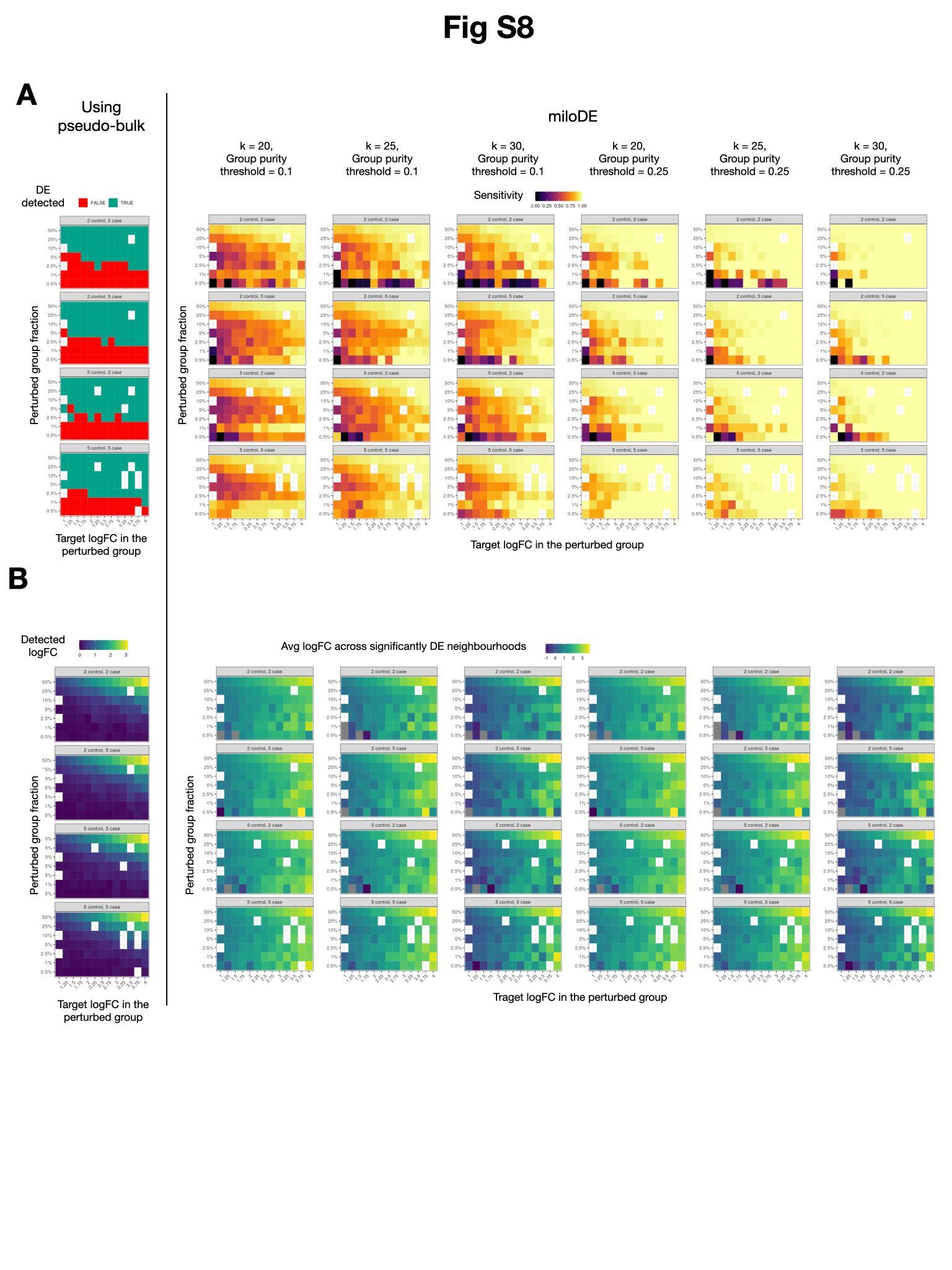
**

**Fig S9. Cells contributing to blood lineage have a higher ‘degree of perturbation’.**

1. UMAP representing a manifold of chimera mouse embryos, colours correspond to the cell types, and facets correspond to whether cells carry a knockout of Tal1.
2. Boxplots representing cell type ranking according to the number of DE genes per neighbourhood, with neighbourhoods being grouped by associated cell type.
3. Boxplots representing cell type ranking according to the number of specifically DE genes (with respect to other neighbourhoods) per neighbourhood, with neighbourhoods being grouped by associated cell type.
4. Barplot representing breakdown by cell types and conditions for the PGC-neighbourhood that shows high ‘degree of perturbation’.

**
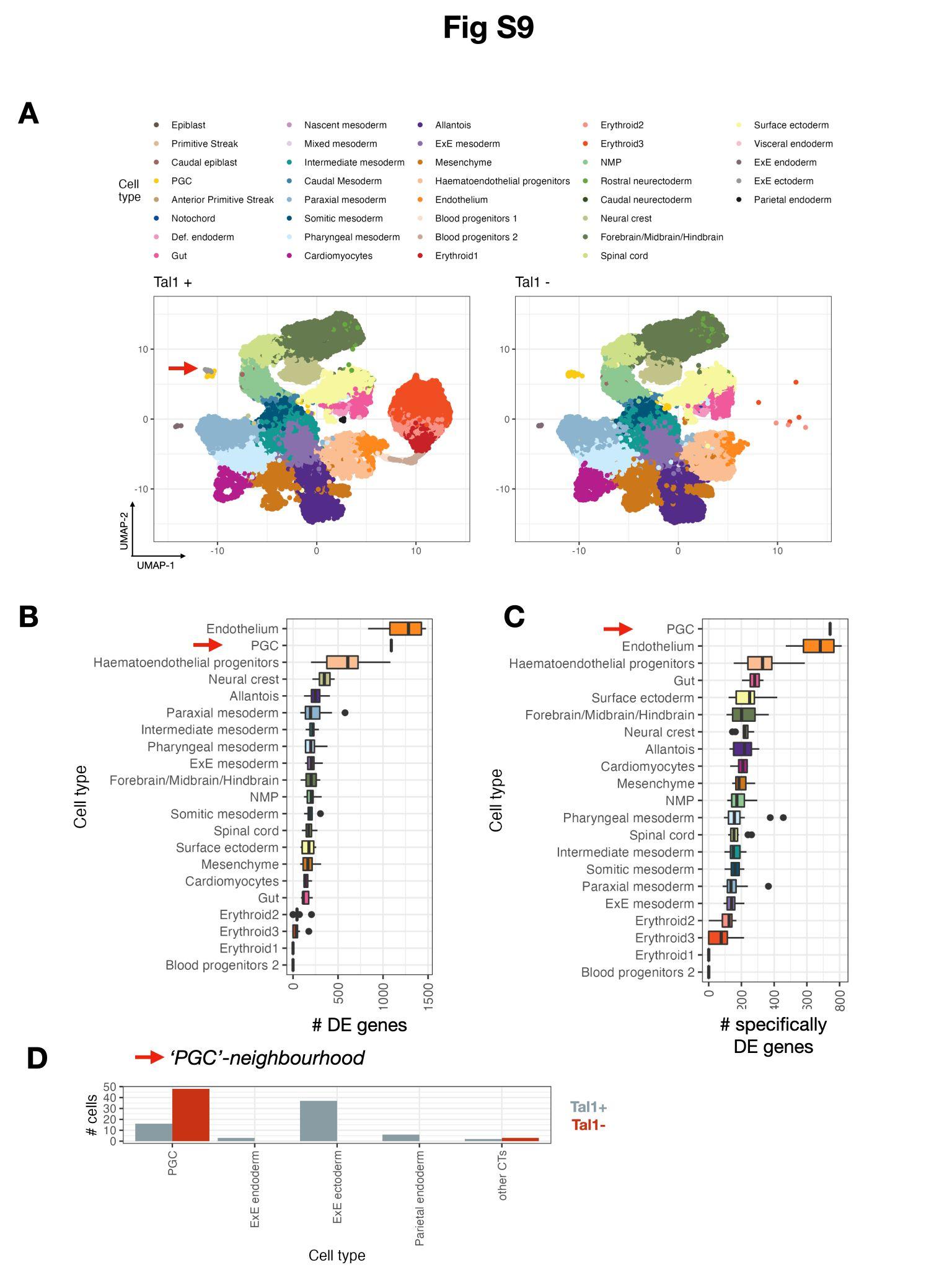
**

**Fig S10. Neighbourhood graphs representing transcriptional profiles for different DE patterns in chimeric mouse embryos lacking Tal1.**

**
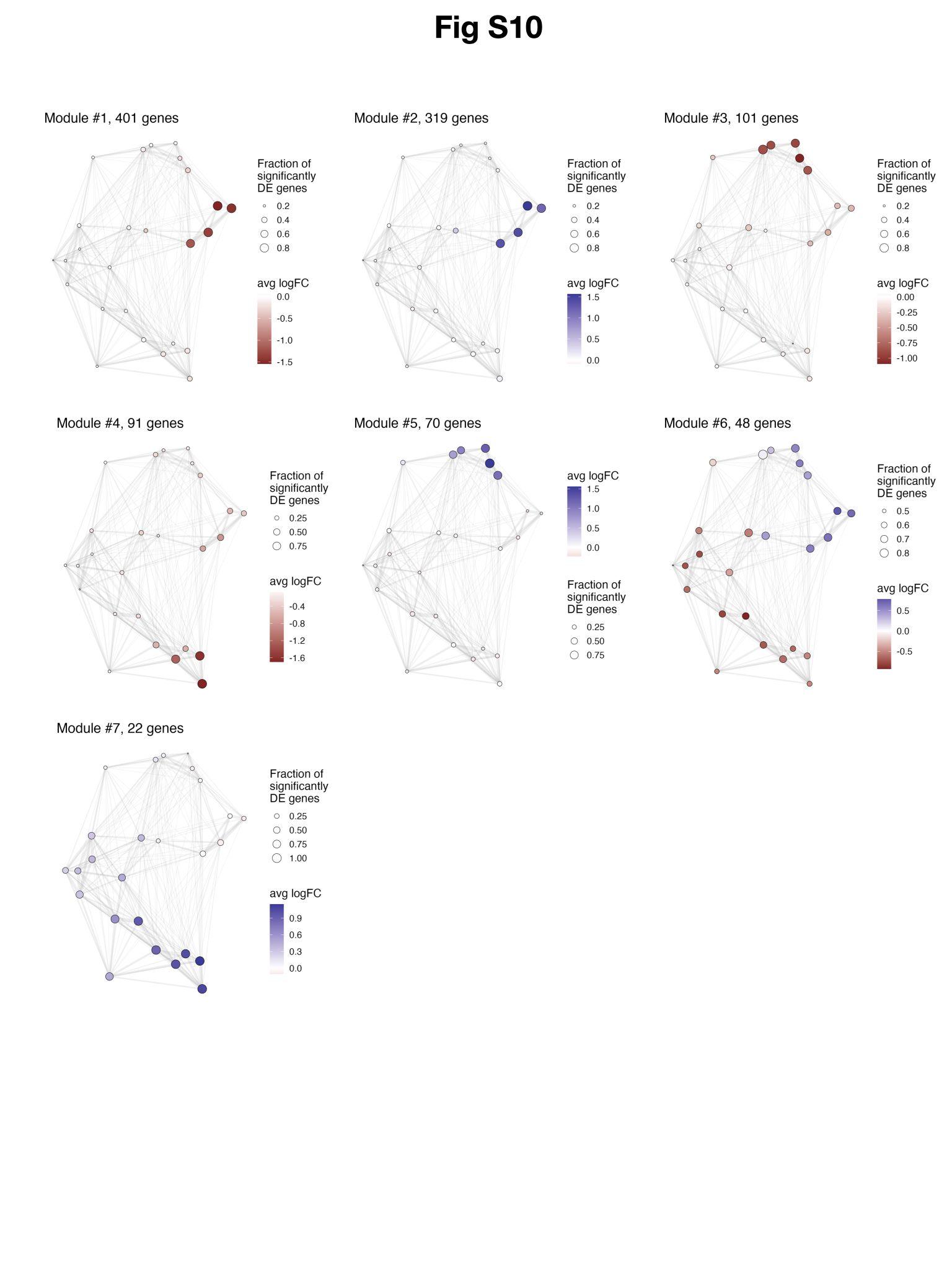
**

**Fig S11. miloDE identifies higher effect size in a specific manner compared to the pseudo-bulk approach.**

1. Boxplot representing logFC distributions across different IPF-upregulated gene sets. Colours correspond to whether a gene has been identified as significantly DE using the pseudo-bulk DE approach (i.e. across all macrophages).
2. Scater plots representing the relationship between logFC using the pseudo-bulk approach (y-axis) and average logFC in significantly DE neighbourhoods using miloDE approach (x-axis). Colours correspond to whether a gene has been identified as significantly DE using the pseudo-bulk DE approach (i.e. across all macrophages).

**
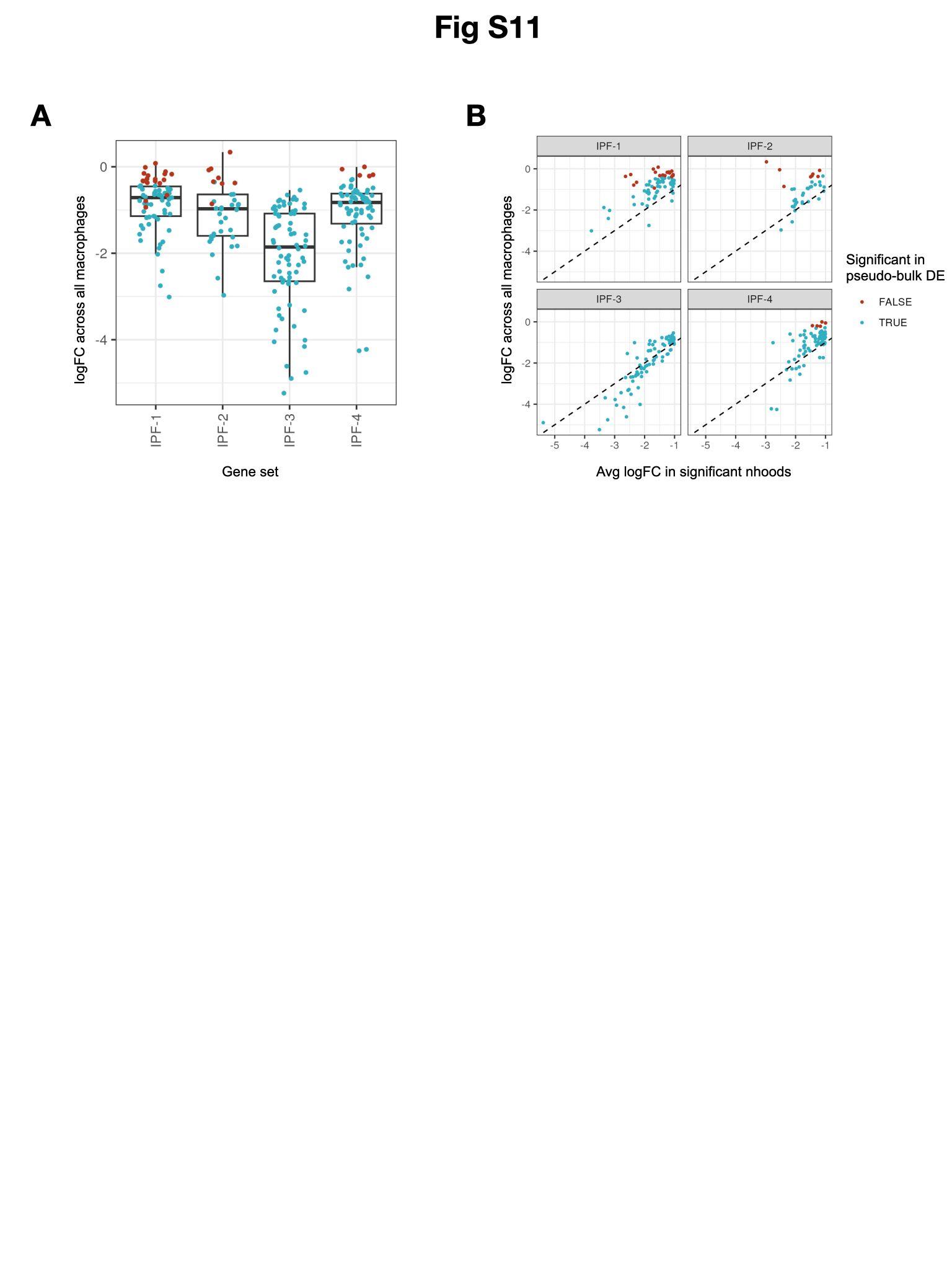
**

**Fig S12. Discrepancies between the pseudo-bulk approach and miloDE are caused by sporadic highly expressed counts and a limited number of cells per neighbourhood tests.**

1. Heatmap representing the breakdown of genes by whether they are detected as DE on the pseudo-bulk level (y-axis) and in how many neighbourhoods they are identified as significantly DE. Colours correspond to log10 of the number of genes in a bin.
2. Boxplots representing the number (top panels) or fraction (bottom panels) of cells with positive expression, either across healthy (left panels) or disease (right panels) cells. The analysis is restricted to genes that are detected as DE in the pseudo-bulk approach, with the detected logFC > 0.5. Genes split into two groups based on whether there are DE neighbourhoods in miloDE analysis (green for true nad red for false). Genes were further binned by the logFC from the pseudo-bulk analyses (x-axis).
3. Top panel: Heatmaps representing breakdown of genes by how many cells they are expressed in (x-axis) and in how many neighbourhoods they are detected as weakly DE (raw p-value < 0.05). The analysis is restricted to genes that are detected as DE in the pseudo-bulk analysis (with logFC > 0.5), however with 0 significantly DE neighbourhoods in the miloDE analysis. Colour corresponds to the number of genes.

Bottom panel: Heatmaps representing average absolute logFC for the corresponding genes.

1. Scater representing co-distribution of logFC (between Spp1-low and Spp1-high cells) when tested within healthy (x-axis) and disease (y-axis) cells. The Red dashed line corresponds to the *x=y* line.
2. Violin plots representing the distribution of logFC (top panels) and -log10(FDR) (bottom panels) for the Spp1-low against Spp1-comparison, either within healthy (left panels) or disease cells (right panels). Genes are binned in the same two groups as in B).

**
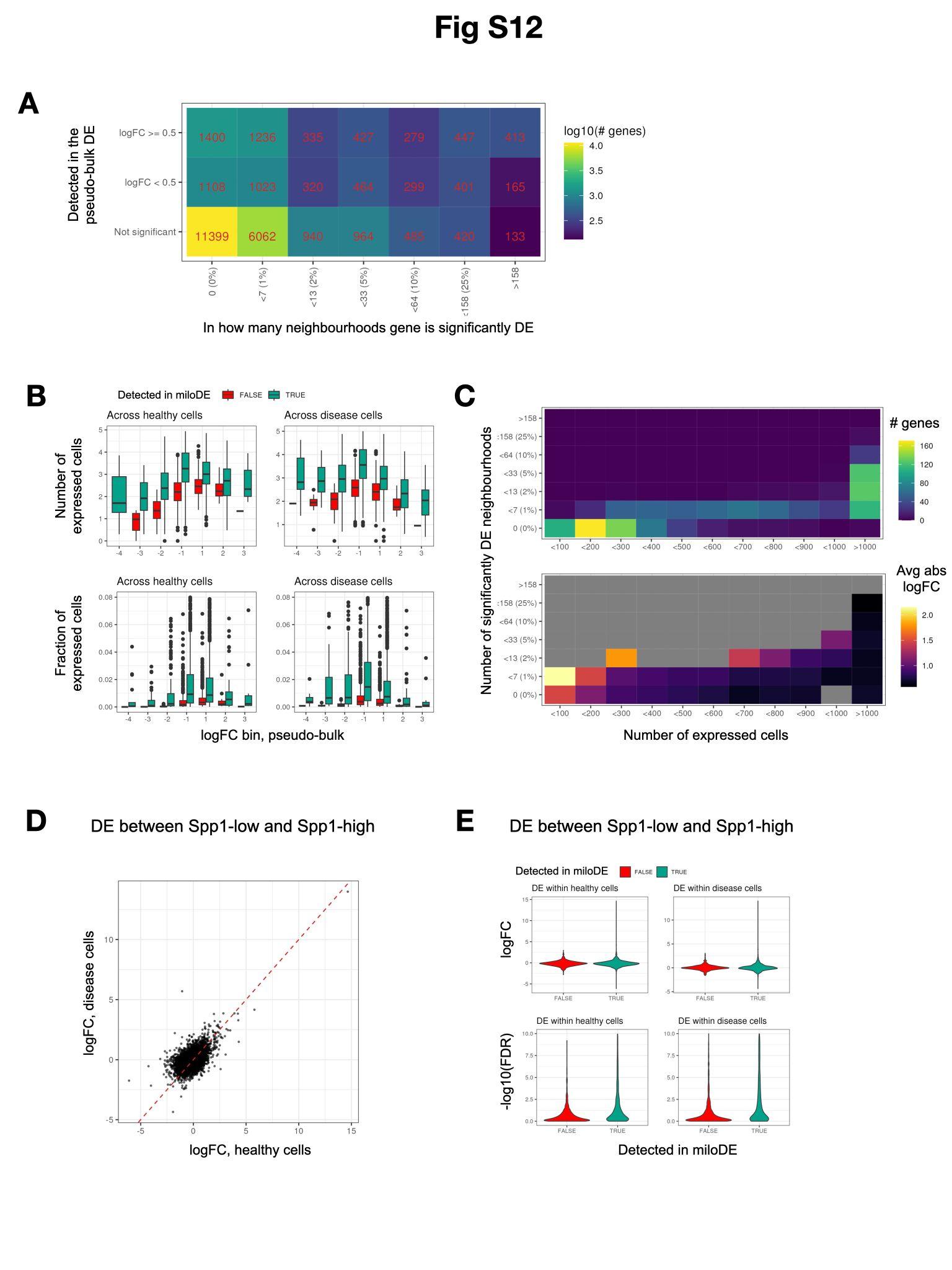
**

**Fig S13. Gene sets vary in their ‘location’ and effect size distributions.**

Scatter plots represent the relationship between logFC, DA (i.e. proxy for phenotypic fibrosis state, x-axis) and average corrected logFC (upper panels) and fraction of significantly DE genes (lower panels). Each point corresponds to one neighbourhood, facets correspond to different gene sets.

**
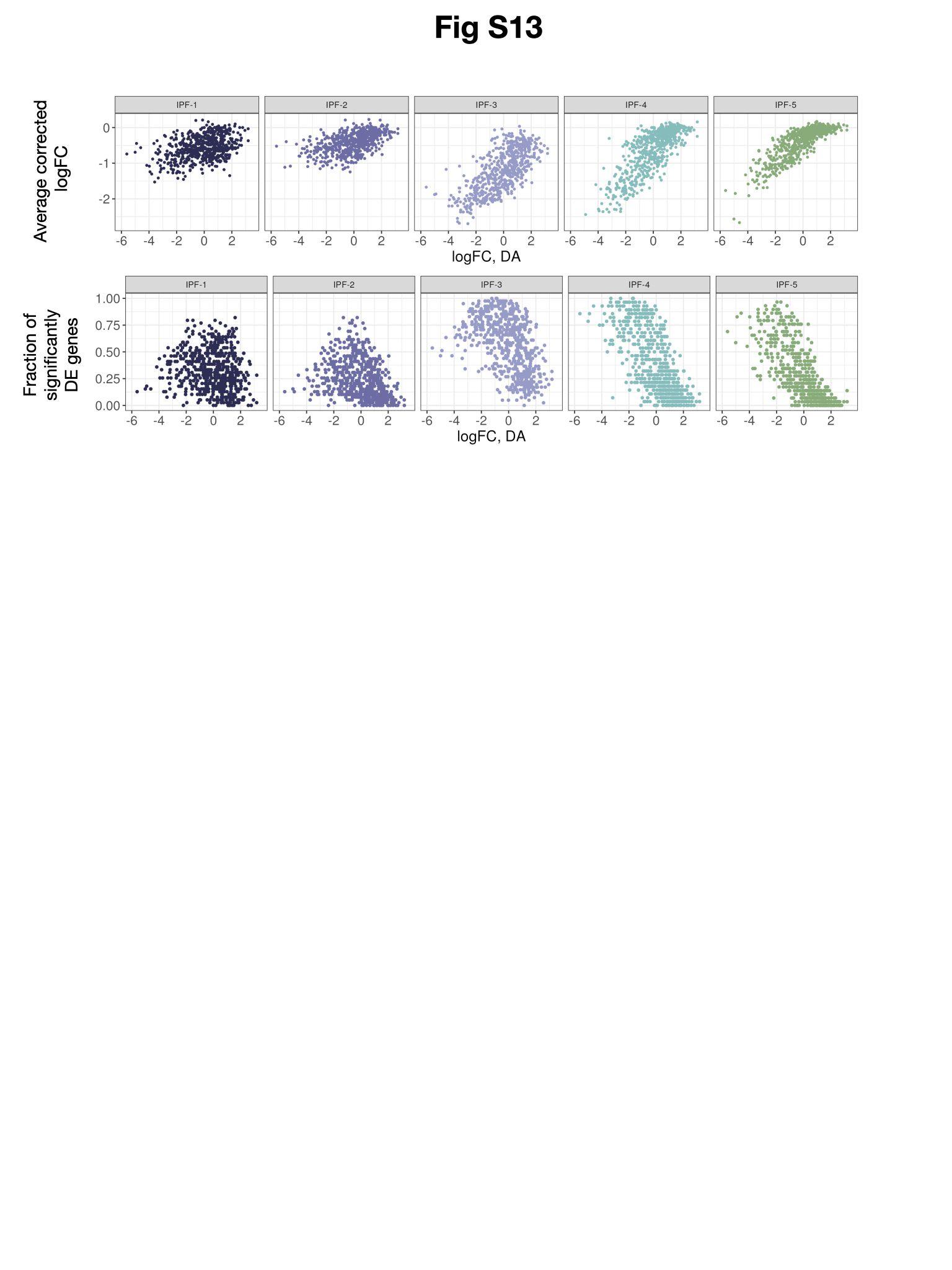
**

**Fig S14. A guidance chart describing all steps of miloDE call and our recommendations for the parameters to enable a sensitive DE detection.**

**
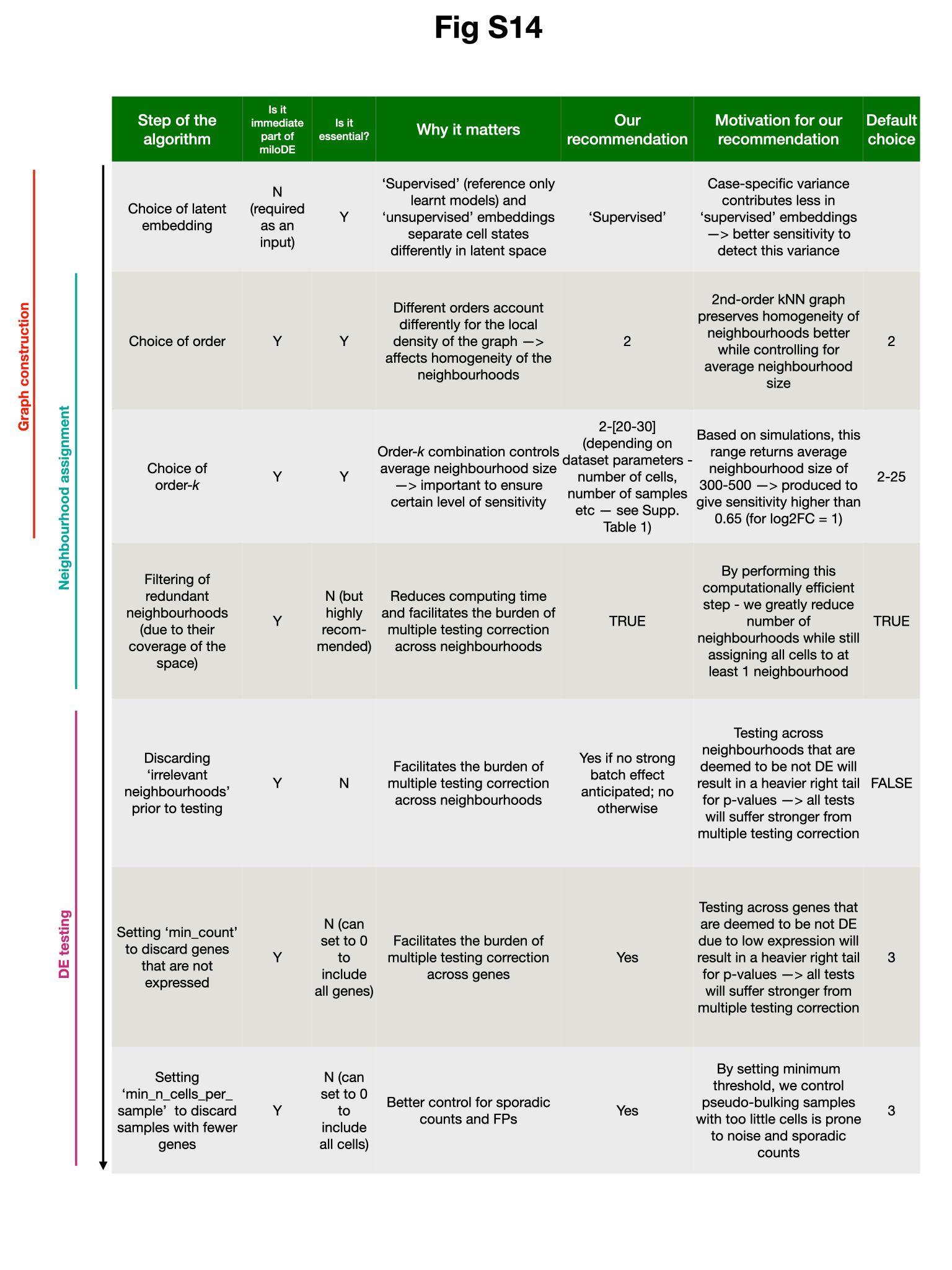
**
